# Microglia in Alzheimer's Disease: It's All About Context

**DOI:** 10.1155/2012/314185

**Published:** 2012-06-20

**Authors:** Tara M. Weitz, Terrence Town

**Affiliations:** ^1^Regenerative Medicine Institute Neural Program and Department of Biomedical Sciences, Cedars-Sinai Medical Center, 8700 Beverly Boulevard, Steven Spielberg Building Room 345, Los Angeles, CA 90048, USA; ^2^Department of Neurosurgery, Cedars-Sinai Medical Center, 8700 Beverly Boulevard, Steven Spielberg Building Room 345, Los Angeles, CA 90048, USA; ^3^Department of Medicine, David Geffen School of Medicine, University of California, Los Angeles, CA 90048, USA

## Abstract

Neuroinflammation is now regarded as both an early event and prime mover in the pathobiology of Alzheimer disease (AD), a neurodegenerative disease that represents a growing public health threat. As the resident innate immune cells within the central nervous system, microglia are centrally positioned as key orchestrators of brain inflammation. It is now accepted that numerous forms of activated microglia exist. Furthermore, while some types of reactive microglia are detrimental, others can actually be beneficial. In the context of AD etiopathology, much debate surrounds whether these enigmatic cells play “good” or “bad” roles. In this article, we distill a complex clinical and experimental literature focused on the contribution of microglia to AD pathology and progression. A synthesis of the literature only seems possible when considering context– the conditions under which microglia encounter and mount immunological responses to AD pathology. In order to carry out these diverse contextual responses, a number of key receptors and signaling pathways are variously activated. It will be critically important for future studies to address molecular mediators that lead to beneficial microglial responses and therefore represent important therapeutic targets for AD.

## 1. Introduction

More than twenty years ago, groundbreaking studies by Wisniewski and his colleagues sparked debate over the relationship between microglia and *β*-amyloid plaques in Alzheimer disease (AD). In their electron microscopic observations of microglia associated with *β*-amyloid deposits in the brain parenchyma and cortical vessel walls of AD patients' brains, Wisniewski and his colleagues discovered that ~80% of a morphologic structure they termed the “amyloid star” was covered by microglia [1, 2]. Although they found that microglia had the ability to phagocytose A*β in vitro*, these innate immune cells appeared in close proximity to amyloid plaques in brains of AD patients but did not contain *β*-amyloid fibrils in their lysosomal compartments. These ultrastructural observations led Frackowiak and coworkers to hypothesize that microglia were unable to phagocytose and remove A*β* *in vivo* and were instead responsible for the manufacture of amyloid fibers [3]. In contrast, Wisniewski did observe *β*-amyloid fibrils in the lysosomes of macrophages that had infiltrated the brain from the periphery in AD patients with rare comorbid stroke [4]—findings that were later confirmed by Akiyama and McGeer [5].

 Rooted in these initial landmark studies of Wisniewski, it is now recognized that there are at least two classes of phagocytic cells responsible for the innate immune responses in the brain—the endogenous microglia and the exogenous hematogenous macrophages [6–8]. Microglia are the brain-resident macrophages derived from monocyte precursors during embryogenesis and provide the initial response to injury from within the central nervous system (CNS). Activation of microglia via neural insults leads to the synthesis of an array of proinflammatory mediators, which can be beneficial for clearing infections and for tissue repair; but, if left uncontrolled, this response can go unresolved and perpetrate bystander neural insult. Thus, in addition to their role as the first responders to CNS injury, activated microglia (and astrocytes) can participate in a form of chronic neuroinflammation termed “reactive gliosis,” which has been implicated in the pathoetiology of a myriad of neurodegenerative diseases. The second class of phagocyte, peripherally-derived macrophages, arises from bone marrow precursors and will be referred to in this review as hematogenous macrophages. These cells can be recruited to the CNS in health and after CNS injury and are primarily believed to traverse the blood-brain barrier (BBB) at postcapillary venules [9]. While hematogenous macrophages typically remain in the perivascular space, they can be recruited into the brain parenchyma by chemokines and cytokines. These proteins are typically released following activation of microglia and astrocytes upon CNS insult and can cause blood-borne mononuclear cells to differentiate into cells that closely resemble brain-resident microglia. Finally, recent focus has been directed towards a third class of brain phagocyte known as the perivascular macrophage. These cells have been found to play an important role in remodeling cerebrovascular A*β* deposits in a transgenic mouse model of cerebral amyloidosis [10].

 Even though the pioneering observations of Wisniewski and his colleagues were made more than two decades ago, it is only recently and with the availability of modern cellular and molecular biology techniques that the roles of brain phagocytes have been more fully interrogated in AD. Still, their significance in the pathoetiology and—even more intriguingly—as a possible treatment modality for AD, remains unclear. What is clear from the studies of the past twenty years, however, is that resident local and peripherally-infiltrating brain macrophages play complex roles in the pathobiology of AD.

## 2. “Bad” versus “Good” Microglia in Alzheimer's Disease

 It has previously been suggested that microglial activation is not simply a single phenotype, and that a continuum exists with antigen presenting cell function (adaptive activation) at one pole and phagocytic cell function (innate activation) at the other [11]. Accordingly, expression profile studies of macrophages in AD and in mouse models of cerebral amyloid also suggest that there is functional heterogeneity in the activation states of microglia that may contribute to disease outcome [12–14]. These innovative concepts support the notion that the context of microglial activation is a key factor in determining what role (whether “bad” or “good”) these enigmatic cells play in AD.

## 3. A Case for “Bad” Microglia

### 3.1. Evidence from Patients

 The concept that microglia primarily play a detrimental role in AD is supported by the Wisniewski studies and by early epidemiologic findings. In general, results from these studies are interpreted as lending support to the idea that activated microglia are likely more harmful than helpful to an AD-afflicted brain. For instance, activated microglia secrete the proinflammatory innate cytokines including tumor necrosis factor-alpha (TNF-*α*) and interleukin-1beta (IL-1*β*), which can directly injure neurons at superphysiologic levels [15, 16].

 In addition to the well-accepted notion that microglia are closely associated with senile plaques in AD, it has also been shown that numerous inflammatory mediators are up-regulated in affected areas of the AD brain [17]. These observations have led to the hypothesis that individuals being treated on a long-term basis with anti-inflammatory medications might be afforded prophylaxis against chronic neuroinflammation and may therefore be at reduced risk for developing AD. In accord with this concept, a number of epidemiologic studies in the early 1990s found that incidence of dementia in elderly patients with arthritis was lower compared to the general population [18–20]. In addition, evidence from neuropathologic studies supports the notion that nonsteroidal anti-inflammatory drugs (NSAIDs; often prescribed for arthritis) reduce microglial activation and thereby dampen brain inflammation [21]. Specifically, Mackenzie and Munoz compared brains from elderly, nondemented arthritic patients with a history of chronic NSAID use and nondemented control subjects that were not NSAID users and found that this latter group had (on average) three-fold more activated microglia than the former. However, interpreting these results is not so straightforward, as arthritis was used as a surrogate for NSAID use in many of these early studies, raising a possible “confounding by indication” issue. Further, The Golde group has shown that a subset of NSAIDs lower A*β*_1−42_, widely considered the neurotoxic A*β* species, independently of cyclooxygenase (COX) activity [22]. Nonetheless, these initial reports prompted more recent work that focused more specifically on NSAID use, and there are now over 25 epidemiologic studies that have shown an inverse risk relationship between NSAID use and AD. A systematic review of these studies uncovered an approximate 50% reduced risk of AD enjoyed by NSAID users compared to nonusers [23].

### 3.2. Evidence from Mouse Models

 The effects of NSAIDs have also been evaluated in transgenic mouse models of cerebral amyloidosis. In the earliest study, Lim et al. tested the effects of 6-month-long treatment of Tg2576 mice with ibuprofen, beginning at 10 months of age when A*β* plaques first appear in these mice. They found that treated animals had significantly reduced amyloid deposition as well as blunted expression of the reactive astrocyte marker glial fibrillary acidic protein (GFAP) and the proinflammatory cytokine, IL-1*β* [24]. Other groups confirmed the ibuprofen results in Tg2576 mice, amyloid precursor protein (APP) plus presenilin-1 (PS1) double transgenics (termed APP/PS1 mice) and APPV717I transgenic mice, all of which showed reduced microglial activation and fewer amyloid deposits following treatment [25–27]. Heneka and colleagues also tested the effects of curcumin, a naturally-occurring NSAID derived from turmeric, which is considered to be a less toxic alternative to COX-2 inhibitors like ibuprofen. They found decreased IL-1*β* and GFAP levels as well as attenuated plaque burden in treated mice [27]. Furthermore, treatment of PS1/APP doubly transgenic mice with the nitric oxide-donating NSAID NCX-2216 reduced amyloid load via a mechanism thought to involve phagocytic microglia [25, 28]. These studies demonstrate that early treatment with NSAIDs can reduce neuroinflammation and amyloid plaque burden in three different transgenic mouse models of cerebral amyloid deposition. However, it should be noted that studies done with the selective COX-2 inhibitor celecoxib failed to reduce inflammatory burden and, in one study, actually increased abundance of A*β*_1–42_ peptide in transgenic AD model mice [25, 29]. Therefore, it seems that certain NSAIDs are capable of reducing microgliosis and amyloidosis in several different transgenic mouse models, while others are not as effective or even produce converse results. This suggests that microglia likely have different responses to such treatment depending on the mechanism of action of the compound and/or the nature of the inflammatory milieu at the initiation of, or even during the course of treatment.

 Studies using mouse genetics as a powerful tool to elucidate the role of microglia in AD pathogenesis have also helped to establish the existence of “bad” microglia that promote disease progression. In early studies done by our group, A*β*-stimulated microglia treated with CD40 ligand (CD40L) showed increased TNF-*α* production and promoted injury of primary cortical neurons. In addition, these studies demonstrated that microglia from transgenic “Swedish” mutant APP (Tg2576) mice deficient for CD40L exhibited reduced activation compared to microglia from CD40L-sufficient transgenic Tg2576 littermates [16]. Moreover, a separate study showed that genetic ablation of CD40L in APP/PS1 mice reduced amyloid plaques and mitigated astrocytosis and microgliosis [30]. Further *in vitro *investigations showed that CD40-CD40L interaction led to decreased microglial phagocytosis of exogenous A*β*_1–42_ and increased production of proinflammatory cytokines. CD40 ligation in the presence of A*β*_1–42_ led to “adaptive” activation of microglia, as evidenced by increased colocalization of major histocompatibility complex class II (MHC II) with A*β*. In addition, when cultured microglia were exposed to A*β*_1–42_ in the presence of CD40L and cocultured with CD4^+^ T cells, T cell-derived interferon-gamma (IFN-*γ*) and interleukin-2 production were stimulated, suggesting that CD40 signaling promotes a microglial proinflammatory antigen presenting phenotype [31]. In the context of CD40-CD40L interaction, these results collectively suggest that microglia become proinflammatory and play a primarily deleterious role in progression of AD-like pathology.

 Additional early evidence for detrimental effects of activated microglia in AD came from Qiao and colleagues. Those authors chronically administered the bacterial endotoxin lipopolysaccharide (LPS) intracerebroventricularly to APPV717F mice that either expressed apolipoprotein E (apoE^+/+^) or were apoE deficient (apoE^−/−^) [32]. While all LPS-treated mice exhibited global astrocytosis and amyloid plaque-localized microglial activation, Qiao et al. found significant acceleration of amyloid deposition in LPS-treated compared to vehicle-treated APPV717F-apoE^+/+^ mice, while this effect was not observed in APPV717F-apoE^−/−^ mice. These experiments suggest that experimental induction of microglial activation by chronic administration of LPS can accelerate amyloidosis in a transgenic mouse model of AD in the presence of apoE, and support the idea that activated microglia exacerbate cerebral amyloidosis.

 More recent studies by Mori and colleagues showed that overexpression of the proinflammatory cytokine-like molecule human S100B (huS100B) in the Tg2576 mouse model of cerebral amyloid deposition resulted in increased parenchymal and cerebrovascular *β*-amyloid deposits and elevated A*β* levels [33]. These effects were accompanied by increased amyloidogenic processing of APP in addition to reactive astrocytosis and microgliosis in Tg2576-huS100B mice. The results by Mori et al. lend further support to the notion that some forms of glial activation (brought about in this case by reactive astrocyte-derived S100B) exacerbate AD-like pathology and are thus likely detrimental to the AD-afflicted brain.

 In addition to proinflammatory cytokines, chemokines play critical roles in orchestrating movement (chemotaxis) of microglia toward noxious stimuli including *β*-amyloid deposits. Recent studies by Fuhrmann and colleagues have shown that knocking out the expression of the fractalkine receptor, Cx3cr1, can prevent neurodegeneration [34]. The expression of Cx3cr1 is exquisitely restricted within the CNS to microglia and is considered to be a critical factor in neuron-microglia communication. Fuhrmann and colleagues used intravital two-photon imaging to observe interactions between microglia and neighboring neurons in 3x Tg-AD mice, which overexpress pathogenic mutant forms of PS1 (M146V), “Swedish” mutant APP, and tau (P301L) [35]. These 3x Tg-AD mice were crossed with a transgenic mouse line expressing yellow fluorescent protein in cortical layers III and V and another line expressing green fluorescent protein in place of the endogenous murine Cx3cr1 locus. Time-lapse intravital imaging showed neuron loss in Cx3cr1-sufficient mice while neurons in Cx3cr1-deficient mice survived. In addition, they observed that Cx3cr1-sufficient microglia rapidly mobilized toward neurons destined for degeneration. However, Cx3cr1-deficient mice did not exhibit change in A*β* abundance, suggesting that the phagocytic ability of microglia in these mice was either not altered or not involved in the observed neuron loss phenotype. Similar studies by Bruce Lamb, Richard Ransohoff, and their colleagues have shown that Cx3cr1 deficiency results in a gene dose-dependent reduction in *β*-amyloid deposition in two different mouse models of AD: APP/PS1 and R1.40 [36]. Interestingly, in their models, Cx3cr1 deficiency also resulted in reduced numbers of microglia surrounding A*β* deposits as well as attenuated immunostaining for CD68 (but not CD45) and altered expression of inflammatory cytokines and chemokines, including reduced levels of TNF-*α* and CCL2 mRNAs, but elevated IL-1*β* mRNA levels. Moreover, the authors demonstrated *in vivo* and *in vitro* that Cx3cr1^−/−^ microglia had enhanced ability to phagocytose A*β*. While the study by Fuhrmann and coworkers did not show differences in A*β* abundance between Cx3cr1-sufficient and -deficient mice, Lee et al. suggested that A*β* aggregates in 3x Tg-AD mice may be more intracellular than extracellular at the age at which this study was done, therefore affecting the ability of microglia to phagocytose these deposits. Nonetheless, these studies suggest that Cx3cr1 signaling plays a role in (a) homing of microglia to neurons that are destined for death and (b) determining microglial responses to cerebral amyloidosis. In both cases, signaling through Cx3cr1 seems to direct microglia to respond in such a way that aids rather than combats the progression of AD-like pathology.

 Finally, previous studies, chiefly from the group of Li-Huei Tsai, have established that activation of cyclin-dependent kinase five (Cdk5) via proteolytic cleavage of p35 to p25 is a key event in AD patient brains that can promote neurodegeneration [37]. However, the role of brain inflammation in this process was not previously clear. In a very recent study, the Kesavapany group examined the consequences of driving p25/Cdk5 activity both *in vivo *and *in vitro* on neuroinflammation and neuronal death [38]. The authors demonstrated that the Cdk5/p25 pathway triggered neuroinflammation via lysophosphatidylcholine that proceeded and led to neurodegeneration and neuronal loss *in vitro*. In addition, they showed that inducible Cdk5/p25 expression *in vivo* caused early neuroinflammation followed by later accumulation of copious intraneuronal A*β*. One interpretation of this finding is that A*β* production signifies an acute-phase stress response that can both be initiated by neuroinflammation and drive it. If this is true, then one could imagine a feed-forward loop endorsing a constellation of damaging inflammatory mediators. These findings provide direct evidence that neuroinflammation, at least as initiated by Cdk5/p25 activation, occurs early and can promote neurodegenerative changes. All of these studies suggesting detrimental actions of microglia are summarized in Table 1.

## 4. A Case for “Good” Microglia

### 4.1. Evidence from Humans and Preclinical Mouse Models

 Inflammatory responses are not always deleterious, and are often even necessary for survival. Given the exquisite sensitivity of neurons to inflammation-induced bystander injury however, there is a fine line between neuroinflammation that results in tissue repair versus excessive damage to brain cells [39]. Epidemiological findings demonstrating reduced risk for AD in patients using NSAIDs led to the design of the Alzheimer disease anti-inflammatory prevention trial (ADAPT), a randomized controlled trial to test the association of NSAID use with cognitive function over time in nondemented elderly individuals. The result of this trial indicated no protection afforded by NSAIDs (naproxen or celecoxib) on cognitive scores, and weak evidence for lower cognitive scores in naproxen users [40, 41]. This latter result suggests that chronic use of naproxen might have actually been detrimental in the ADAPT study. If correct, there exists a possibility that microglial activation was actually beneficial and that treatment with naproxen could have negated this effect. While these results need to be interpreted with caution since the trial was prematurely halted due to fear of cardiotoxicity associated with certain NSAIDs, other trials testing NSAIDs for treatment of AD or as preventative agents for mild cognitive impairment also had null findings [42]. That the clinical studies did not support epidemiological findings raises the possibility that the context of microglial activation and other factors such as whether individuals were cognitively healthy or “on the verge” of conversion to mild cognitive impairment or to AD at the time of NSAID treatment are critically important outcome determinants [43]. Also, because many of the healthy elderly participants in the ADAPT trial were arthritics, these individuals' peripheral immune environment may impact their risk for later developing AD [44] and therefore could represent a confound to interpreting the risk relationship between NSAID use and development of AD.

 While the above reports investigated the effects of NSAIDs in the clinic, studies in the late 1990s from Dale Schenk and colleagues at Elan pharmaceuticals yielded surprising results in preclinical mouse models. They administered peripheral injections of A*β*_1–42_ plus complete Freund's adjuvant into young PDAPP transgenic mice, which overexpress mutant APP. Their intention was to worsen AD-like pathology in these mice. However, they instead found that A*β*_1–42_ plus adjuvant given to young mice essentially blocked *β*-amyloid plaque formation, and treatment of older animals significantly mitigated the extent of cerebral amyloid deposits. In addition, Schenk and colleagues observed MHC II-positive microglia colocalized with A*β* plaques in A*β*_1–42_ treated mice, which were not observed in control mice treated with PBS plus adjuvant [45]. These results were supported by independent studies that demonstrated decreased behavioral impairment in A*β*-immunized PDAPP mice [46, 47]. In a subsequent study, Bard and colleagues showed that passive transfer of A*β* antibodies from vaccinated mice to transgenic PDAPP mice also reduced cerebral amyloidosis. In that study, they observed punctate immunoreactivity for A*β* that co-localized with activated microglia, and suggested that A*β* plaque clearance was mediated by antibodies against amyloid *β*-peptide that crossed the BBB and triggered microglial cells through Fc receptor-mediated phagocytosis [48]. While microglia are generally regarded as inefficient A*β* phagocytes [11], Bard and colleagues suggested the passive A*β* vaccine was somehow able to lure microglia into phagocytosing A*β* antibody-opsonized plaques.

 Based on encouraging results in mouse models, Elan pharmaceuticals and Wyeth partnered to develop an A*β* vaccine for use in humans. The drug, named AN-1792, consisted of synthetic A*β*_1–42_ peptide in QS-21 adjuvant, and a phase I trial did not reveal significant adverse effects in a limited cohort of 80 subjects. The phase IIa trial was halted prematurely, however, when four participants (~5-6% of study subjects) developed clinical signs consistent with aseptic meningoencephalitis [49]. Shortly after the trial was halted, a case report was published of a 72-year-old woman who had a history of probable AD and had received AN-1792 and responded with elevated A*β* antibody titers. Upon histological examination, her brain showed extensive areas of the neocortex with very few plaques and regions devoid of plaques that had punctate immunoreactivity for A*β* that often colocalized with phagocytic microglia. Such results were consistent with previously published reports in AD transgenic mice suggesting that the vaccine instigated microglial clearance of A*β* [50]. A subsequent study of eight participants who received immunization and developed A*β*-specific antibodies showed clear evidence of amyloid plaque removal but no effect of immunotherapy on prevention of cognitive decline [51]. Together, these data suggest that positive signal in mouse models of cerebral amyloidosis does not always translate to human AD and, in the case of A*β* immunotherapy, that careful preclinical toxicology in non-human primates is critically important.

### 4.2. Evidence from Mouse Genetics Approaches

 Previous studies have shown elevated CD45 expression on reactive microglia in AD brains compared with controls [52], and our group investigated the role of CD45 in responsiveness of microglia to A*β* peptides [53]. Because CD45 is a membrane-bound protein-tyrosine phosphatase, we inhibited its function in the context of A*β* stimulation by cotreating primary cultured microglia with a tyrosine phosphatase inhibitor and A*β* peptides. This resulted in secretion of TNF-*α* and nitric oxide that injured neurons in coculture conditions. Furthermore, treatment with an agonistic CD45 antibody markedly inhibited these detrimental effects via blocking p44/42 mitogen-activated protein kinase, suggesting that CD45 activation promotes beneficial, neuroprotective function of microglia. After stimulation with A*β* peptides, primary cultured microglia from CD45-deficient mice exhibited copious TNF-*α* release, nitric oxide production, and neuronal injury, and brains from Tg2576 mice deficient for CD45 had significantly increased production of TNF-*α* compared with CD45-sufficient Tg2576 littermates. In more recent studies, we reported that CD45-deficient PSAPP mice had increased abundance of soluble oligomeric and insoluble A*β* (both extracellular and intracellular species), increased TNF-*α* and IL-1*β* proteins, and neuronal loss compared with CD45-sufficient PSAPP littermates [54]. These studies demonstrate that the cell surface marker CD45 promotes “good” microglial activation in the context of A*β* challenge, likely by endorsing an A*β* phagocytic phenotype that mitigates cerebral amyloidosis.

 There are also studies demonstrating that proinflammatory cytokines can promote beneficial neuroinflammation that actually resolves cerebral amyloidosis in transgenic mice. In one of the earliest reports, Shaftel and colleagues studied the role of IL-1*β* in chronic neuroinflammation and in AD by engineering an IL-1*β* ^XAT^ transgenic mouse [55]. This model was constructed to overexpress IL-1*β* in the CNS using the GFAP promoter. Following injection of the FIV-Cre construct into hippocampi of these mice, a STOP codon in the IL-1*β* ^XAT^ transgene is excised, thus activating overexpression of IL-1*β* in a temporal and spatial manner. In this mouse model, IL-1*β* expression led to robust neuroinflammation characterized by activation of astrocytes and microglia and induction of proinflammatory cytokines. Moreover, when IL-1*β* ^XAT^ mice were crossed with the APP/PS1 mouse model of AD and the hippocampi of the resulting compound transgenic mice were injected with FIV-Cre, those authors observed dramatically reduced amyloid plaque pathology in the injected area of the brain. Additionally, Shaftel and colleagues showed that IL-1*β* overexpression caused an increase in the number of microglia overlapping with amyloid plaques, an increase in Iba1 staining intensity, and high levels of MHC II expression in the same cells. These findings implicate IL-1*β* expression in activating a “good” form of neuroinflammation in APP/PS1 mice, which the authors suggested mediated enhanced phagocytosis of amyloid plaques by activated microglia. However, their studies do not rule out the role that other cell types might have played in this scenario.

 In another study, Chakrabarty and colleagues used recombinant adeno-associated virus serotype 1 (rAAV1) to express murine IFN-*γ* (mIFN-*γ*) in brains of the TgCRND8 mouse model of cerebral amyloidosis. Those authors demonstrated the ability of this potent proinflammatory cytokine to clear amyloid plaques [56]. Specifically, neonatal TgCRND8 mice injected with rAAV1-mIFN-*γ* in cerebral ventricles were euthanized at 3 months of age for analysis. The results showed widespread increased immunoreactivity for both GFAP and Iba1 in their brains. In addition, brains of these mice exhibited decreased levels of soluble A*β* and A*β* plaque burden, and did not show evidence of altered APP processing. Similar results were found after rAAV1-mIFN-*γ* injection into hippocampi at 4 months of age and pathological analysis 6 weeks later. Furthermore, mIFN-*γ* expression *in vivo* resulted in significant up-regulation of several microglial markers (e.g., MHC I, MHC II, CD11b, and CD11c), suggesting a microglial phenotype reminiscent of an antigen-presenting cell. Chakrabarty and colleagues also demonstrated in *in vitro* studies that mouse microglia primed with mIFN-*γ* had increased uptake of fluorescently tagged A*β*_1–42_ aggregates compared to control microglia. These results were not only restricted to mIFN-*γ*, as similar studies from this group using IL-6 and TNF-*α* rAAV approaches produced consistent findings [57, 58]. In contrast to the findings of Qiao et al. outlined in the previous section, DiCarlo and colleagues found that induction of multiple proinflammatory molecules following a single intrahippocampal injection of LPS into APP/PS1 transgenic mice resulted in activated microglia but reduced A*β* plaque load compared to saline-injected mice [59]. When taken together, these results suggest that certain forms of proinflammatory microglial activation are potentially beneficial for reducing AD-like pathology in transgenic mouse models.

 In a different experimental paradigm, Wilcock and colleagues deleted the microglial proinflammatory nitric oxide synthase 2 (NOS2) gene in the APPSwDI mouse model of cerebrovascular amyloidosis. Those authors were able to create a model with progressive amyloid pathology as well as tau pathology and neuronal loss [60]. The authors pointed out that a difference in nitric oxide (NO) production between human and mouse microglia may be a key factor in this result. As such, they hypothesized that lower levels of NO production by human microglia create an environment in which AD-like pathology is endorsed, whereas high levels of NO (such as those produced by mouse microglia) are neuroprotective. Therefore, the authors suggest that NOS2 deficiency creates a milieu that is conducive to AD pathology. This study provides yet another interesting example of how the context of the brain inflammatory milieu can determine whether microglia play a beneficial or detrimental role in the progression of AD-like pathology. All of the studies reviewed suggesting beneficial actions of microglia are summarized in Table 2.

## 5. Heterogeneous Microglial Activation States

 As underscored by the studies reviewed above, it is becoming more and more appreciated that microglial activation is not simply a single phenotype. In fact, the most parsimonious interpretation of the evidence thus far points to broad heterogeneity of microglial activation states. But an open question remains as to how to best define these various forms of reactive microglia. On the one hand, an activation “continuum” likely exists that makes delimiting discrete phenotypes difficult [61]. A complementary view is that microglia exist in at least three distinct activated forms in the context of neuroinflammatory diseases: classical (proinflammatory) activation, alternative (anti-inflammatory) activation, and acquired deactivation [62, 63]. In the context of mouse models of cerebral amyloidosis, it is now clear that microglia exhibit a mixed pattern of activation, with elements of both classical and alternative activation [62]. The picture is further complicated by different populations of microglia likely coexisting, each population with its own activated phenotype. Clearly, one of the great challenges for the field will be to define robust and informative markers that predict functional outcomes of these heterogeneous microglial activation phenotypes.

## 6. But Do Brain-Resident Microglia Play Any Role at All?

 While many of the studies cited above suggest that microglia can be deleterious or beneficial in the context of AD-like pathology depending on contextual cues, a recent study suggests that brain-resident microglia do not play a significant role in the formation, maintenance or clearance of amyloid plaques. Mathias Jücker and colleagues used a CD11b-tyrosine kinase/ganciclovir suicide gene approach to kill microglia for two to four weeks in two different AD mouse models (APP/PS1 and APP23) [64]. Selective ablation of microglia in APP/PS1 transgenic mice did not result in differences in total A*β* burden, plaque morphology, or distribution of cerebral A*β* deposits. While it is possible that ablation of brain-resident microglia for two to four weeks is not sufficient time to observe a significant change in A*β* plaque remodeling in transgenic mouse models of cerebral amyloid deposition, this study implies that microglia are not always primary players in the complex landscape of AD pathobiology.

## 7. Hematogenous Macrophages

 As mentioned earlier, CNS-exogenous hematogenous macrophages have been suggested to play a role in the innate immune responses in the brain. Independent studies from the laboratories of Mathias Jücker and Serge Rivest have shown limited infiltration of peripheral mononuclear cells into the CNS of cerebral amyloidosis mouse models [65, 66]. Both studies demonstrated that bone marrow-derived cells were spatially associated with amyloid plaques. While Stalder and coworkers were not able to uncover evidence of amyloid phagocytosis within these cells by electron microscopy, Simard and colleagues presented convincing evidence of amyloid deposits in GFP^+^ infiltrating mononuclear phagocytes. Further, these authors crossed APP/PS1 mice with CD11b-tyrosine kinase mutant (TK^mut30^) transgenic mice in which proliferating CD11b^+^ cells can be specifically ablated by administering the antiviral drug ganciclovir. Using this mouse model, the authors were able to convincingly show that proliferating MHC II-positive peripheral mononuclear phagocytes played an important role in restricting *β*-amyloid plaques. Another landmark study in this area was from the group of Joseph El Khoury. By crossing mice deficient in the chemokine receptor Ccr2 with the Tg2576 mouse model of cerebral amyloidosis, they were able to demonstrate that restriction of hematogenous microglia entry into the brain led to increased plaque load [67].

 These observations led to studies by our group investigating whether peripheral mononuclear phagocytes (hematogenous macrophages) could be targeted to infiltrate into the brain and militate against AD-like pathology. We engineered a CD11c promoter-driven dominant-negative transforming growth factor-beta (TGF-*β*) type II receptor transgene in C57BL/6 mice (CD11c-DNR mice) [68], and were thus able to genetically interrupt TGF-*β* and downstream Smad 2/3 signaling specifically on peripheral innate immune cells. We crossed CD11c-DNR mice with the Tg2576 mouse model of cerebral amyloid and evaluated behavioral impairment and AD-like pathology [69]. Interestingly, doubly transgenic mice showed up to 90% attenuation of brain parenchymal and cerebrovascular amyloid deposits. Further, these animals exhibited partial amelioration of cognitive impairment and reduced astrocytosis, effects that were associated with increased infiltration of A*β*-containing peripheral mononuclear phagocytes in and around cerebral vessels and amyloid plaques. We were also able to observe A*β* immunoreactivity within the cytoplasm of these cells, suggesting that these peripheral macrophages were actively clearing A*β* via phagocytosis. In addition, the peripherally-derived macrophages displayed an anti-inflammatory CD45^+^CD11b^+^Ly-6C^−^ cell surface phenotype and secreted elevated levels of the canonical anti-inflammatory cytokine, interleukin-10 [70, 71], suggesting that the beneficial effect of reduced *β*-amyloid did not come at the cost of increased brain inflammation. Additionally, we were able to demonstrate ~3-fold increased phagocytosis of A*β* by CD11c-DNR versus wild-type macrophages *ex vivo* and similar effects *in vitro* with pharmacologic inhibitors of TGF-*β*-Smad2/3 signaling, suggesting that inhibition of TGF-*β*-Smad 2/3 signaling allows for both peripheral mononuclear phagocyte recruitment to brains of AD model mice and for phagocytic amyloid removal.

## 8. Concluding Remarks

**Figure 1:**
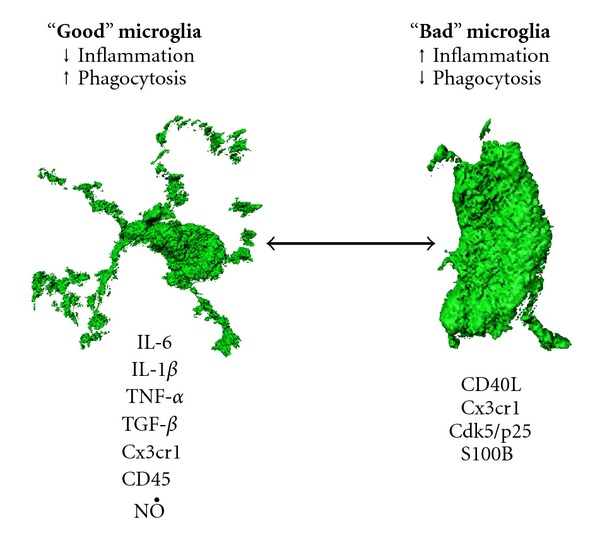
“Good” versus “Bad” microglia in Alzheimer disease. Illustration using 3D models of ramified “good” microglia and ovoid “bad” microglia along with the array of immune molecules that likely help to determine which activated form of microglia will respond to AD-like pathological lesions. It is postulated that microglial phenotype can interconvert between ramified and ovoid functional states. The 3D images were generated using Imaris: Bitplane 3D modeling software and are provided courtesy of David Gate.

 It is now becoming widely appreciated that there are numerous forms of “activated” microglia, some of which are detrimental, and others, beneficial (Figure 1). In this paper, we have attempted to survey the state of the field with respect to both “good” and “bad” forms of reactive microgliosis in terms of AD-like pathology. A synthesis of the literature only seems possible when considering context—the conditions under which microglia encounter AD-like pathological lesions. Specifically, a model emerges where microglia mount different types of activated responses depending on whether they encounter particular species of misfolded protein (A*β* or perhaps even tau) and whether this innate recognition occurs early on or after pathology is well-established. While it is not yet clear how microglial cells differentiate between these various forms of pathogenic peptides and proteins, it is likely that a varied set of immune molecules orchestrate these complex innate immune responses. A deeper understanding of these molecules, and specifically, which pathways lead to beneficial responses to clear pathogenic misfolded proteins in AD, will be key for harnessing these innate immune cells to militate against neuropathology.

## Figures and Tables

**Table 1:** “Bad” microglia in Alzheimer disease.

Publication(s)	Type of Study	Observations
Wisniewski et al., 1989 [1], 1992 [2]; Frackowiak et al., 1992 [3]	Neuropathologic	Microglia are “frustrated phagocytes” responsible for manufacture of amyloid fibrils and not for their removal.
Meda et al., 1995 [15]	*In vitro*	Activated microglia secrete proinflammatory cytokines that promote neural injury at high levels.
Tan et al., 1999 [16]	Mouse models	A*β* and CD40L-stimulated microglia release TNF-*α* that injures primary cortical neurons. CD40 ligand-deficient Tg2576 mice have reduced microglial activation and tau hyperphosphorylation.
McGeer et al. 1990 [18], 1992 [19], 1996 [20]	Epidemiologic	There is lower incidence of dementia in elderly patients with arthritis compared to the general population.
Mackenzie and Munoz, 1998 [21]	Neuropathologic	Chronic NSAID users with senile plaques have 3-fold less activated microglia than non-users.
Szekely et al., 2004 [23]	Epidemiologic	Systematic review of over 25 epidemiologic studies shows ~50% reduced risk of AD associated with NSAID use.
Lim et al., 2000 [24], 2001 [72]	Mouse models	NSAID-treated Tg2576 mice have significantly reduced amyloid deposition, astrogliosis, and IL-1*β* abundance.
Jantzen et al., 2002 [25]; Yan et al., 2003 [26]; Heneka et al., 2005 [27]	Mouse models	Ibuprofen-treated Tg2576, APP/PS1 or APPV717I transgenic mice have reduced microglial activation and amyloid deposits.
Tan et al., 2002 [30]	Mouse models	Genetic or pharmacologic ablation of CD40 ligand in Tg2576 mice reduces cerebral amyloidosis and mitigates gliosis.
Townsend et al., 2005 [31]	*In vitro*	CD40 ligand in the presence of A*β*_(1–42)_ promotes a microglial proinflammatory antigen presenting cell phenotype.
Qiao et al., 2001 [32]	Mouse models	Chronic intracerebroventricular injection of LPS accelerates A*β* plaque load in APPV717F transgenic mice.
Mori et al., 2010 [33]	Mouse models	Forcing expression of proinflammatory S100B accelerates glial activation and cerebral amyloid pathology in Tg2576 mice.
Fuhrmann et al., 2010 [34]	Mouse models	Cx3cr1 endorses microglial-mediated neuronal loss in 3xTg AD mice.
Lee et al., 2010 [36]	Mouse models	Cx3cr1 deficiency reduces cerebral amyloid and reactive microglia in APP/PS1 and R1.40 mice. Cx3cr1^−/−^ microglia have greater A*β* uptake.
Sundaram et al., 2012 [38]	Mouse models	Neuroinflammation occurs early and promotes neurodegeneration mediated by lysophosphatidylcholine and Cdk5/p25. Inducible p25 expression *in vivo* triggers neuroinflammation and intraneuronal A*β*.

**Table 2:** “Good” microglia in Alzheimer disease.

Publication(s)	Type of study	Observations
Martin et al., 2008 [41]	Epidemiologic	No primary prevention of AD in an NSAID clinical trial; weak evidence for lower cognitive scores in naproxen users.
Schenk et al., 1999 [45]	Mouse models	A*β* immunotherapy mitigates *β*-amyloid plaque pathology in PDAPP mice; phagocytic microglia colocalize with remaining A*β* deposits.
Janus et al., 2000 [46]; Morgan et al., 2000 [47]	Mouse models	Behavioral impairment is reduced in A*β*-immunized TgCRND8 or APP/PS1 transgenic mice.
Bard et al., 2000 [48]	Mouse models	Passive A*β* immunotherapy reduces cerebral amyloidosis in PDAPP mice. Remaining A*β* deposits colocalize with phagocytic microglia.
Nicoll et al., 2003 [50]	Neuropathologic	AN-1792 A*β* immunotherapy results in striking reduction of amyloid pathology; remaining plaques colocalize with phagocytic microglia.
Tan et al., 2000 [53]	Mouse models	Microglial CD45 negatively regulates microglial activation and opposes activated microglia-induced neuronal cell injury.
Zhu et al., 2011 [54]	Mouse models	Microglial CD45 endorses phagocytosis and clearance of A*β* peptides and oligomers in PSAPP mice.
Shaftel et al., 2007 [55]	Mouse models	Forcing brain IL-1*β* expression using IL-1*β* ^XAT^ transgenic mice activates “good” neuroinflammation and dramatically reduces amyloid plaque pathology in APP/PS1 mice.
Chakrabarty et al., 2010 [56]	Mouse models	Forcing cerebral IFN-*γ* expression leads to profound neuroinflammation and clears amyloid plaques in TgCRND8 mice via microglial phagocytosis.
Chakrabarty et al., 2010 [57]	Mouse models	Overexpressing brain IL-6 leads to massive gliosis and clears amyloid plaques in TgCRND8 mice via microglial phagocytosis.
Chakrabarty et al., 2011 [58]	Mouse models	Increasing cerebral proinflammatory TNF-*α* expression clears amyloid pathology in TgCRND8 mice.
DiCarlo et al., 2001 [59]	Mouse models	Acute intrahippocampal injection of LPS reduces A*β* plaque load in APP/PS1 transgenic mice.
Wilcock et al., 2008 [60]	Mouse models	APPSwDI mice deficient in proinflammatory NOS2 have tau pathology and neuronal loss.
